# Impact of obesity on the CCR6-CCL20 axis in epidermal γδ T cells and IL-17A production in murine wound healing and psoriasis

**DOI:** 10.1093/jimmun/vkae011

**Published:** 2025-01-23

**Authors:** William Lawler, Tanya Castellanos, Emma Engel, Cristian R Alvizo, Antolette Kasler, Savannah Bshara-Corson, Julie M Jameson

**Affiliations:** Department of Biological Sciences, California State University San Marcos, San Marcos, CA, United States; Department of Biological Sciences, California State University San Marcos, San Marcos, CA, United States; Department of Biological Sciences, California State University San Marcos, San Marcos, CA, United States; Department of Biological Sciences, California State University San Marcos, San Marcos, CA, United States; Department of Biological Sciences, California State University San Marcos, San Marcos, CA, United States; Department of Biological Sciences, California State University San Marcos, San Marcos, CA, United States; Department of Biological Sciences, California State University San Marcos, San Marcos, CA, United States

**Keywords:** diabetes, inflammation, psoriasis, skin, T cells

## Abstract

Obesity is associated with comorbidities including type 2 diabetes, chronic nonhealing wounds, and psoriasis. Normally, skin homeostasis and repair is regulated through the production of cytokines and growth factors derived from skin-resident cells including epidermal γδ T cells. However, epidermal γδ T cells exhibit reduced proliferation and defective growth factor and cytokine production during obesity and type 2 diabetes. One of the genes modulated in epidermal γδ T cells during obesity and type 2 diabetes is CCR6, which is the receptor for CCL20. CCL20 is elevated in the skin during obesity and type 2 diabetes. Here, we identify a subset of murine epidermal γδ T cells that express CCR6 upon activation, both in vitro and in vivo. We show that CCL20 stimulates epidermal γδ T cells to produce interleukin (IL)-17, indicating that CCR6 regulates the IL-17 axis in epidermal γδ T cells. In murine models of wound repair and psoriasis, these epidermal γδ T cells upregulate CCR6 and produce IL-17, with obesity amplifying this response during wound repair but having less effect during psoriasis. These findings have novel implications for the regulation of a specific population of IL-17–producing epidermal γδ T cells during skin damage and inflammation.

## Teaser

- The Editors have selected this article as a highlight of the issue.

## Introduction

Obesity is correlated with increases in skin comorbidities, including chronic nonhealing wounds, which affect 2.5% of the U.S. population, and psoriasis, which affects 8 million Americans.^1^^,^^2^ Normally the skin provides a protective barrier from mechanical, chemical, and pathogenic external threats. The tumor necrosis factor α (TNF-α)/interleukin (IL)-17 axis is key to this protection, as it provides antimicrobial roles and induces keratinocyte proliferation and neutrophil responses in wound repair.^3^^,^^4^ However, dysregulation of the TNF-α/IL-17 axis caused by obesity alters the cellular composition and function in the skin, resulting in premature keratinocyte differentiation, altered skin-resident T cell number and function, and increased barrier permeability.^4^^,^^5^ All of these factors negatively impact chronic nonhealing wounds and psoriasis.^1^

Alterations in TNF-α and IL-17 production during wound repair and psoriasis have been attributed to dermal γδ T cells, dermal Th17 αβ T cells, innate lymphoid cells, and mucosal-associated invariant T cells.^6–9^ In contrast, resident epidermal γδ T cells, also known as dendritic epidermal T cells, have been largely considered bystanders and not active IL-17 producers.^10^ Epidermal γδ T cells bear the Vγ5Vδ1 T cell receptor (TCR) and are rapidly activated by stressed or damaged keratinocytes to release cytokines, chemokines, and growth factors, including KGF-1 and, in some cases, IL-17A.^11–13^ Unlike dermal Vγ6 or Vγ4 T cells, epidermal γδ T cells arise first during T cell development in the fetal thymus and require Skint-1.^14^^,^^15^ In addition, epidermal γδ T cells exhibit distinct functions from dermal γδ T cells including rapid production of KGFs during wound repair and IGF-1 during skin homeostasis.^16–19^ Obesity causes a reduction in cytokine and growth factor production by epidermal γδ T cells at the wound site indicating a shift in function.^12^ Because epidermal γδ T cells normally act early in wound repair, they may also negatively impact chronic wounds and inflammatory skin disease.^10^

One receptor that has been associated with IL-17–producing dermal γδ T cells (Tγδ17) is CCR6. The majority of murine dermal Vγ4 and Vγ6 T cells express CCR6, which facilitates recruitment to the epidermis in response to psoriasis-like inflammation.^10^^,^^20^^,^^21^ CCR6 is also required for efficient wound repair, as CCR6^−/−^ mice exhibit delays in wound closure.^22^ Obese and diabetic patients exhibit elevated CCL20 in the skin, which likely impacts CCR6^+^ T cell recruitment and function.^23^ Although CCR6 regulates other IL-17–producing T cell populations, epidermal γδ T cells have not been included, as they already reside in the epidermis and do not express CCR6 constitutively.^10^^,^^24^ Thus, epidermal γδ T cells may exhibit unique regulation and function of CCR6, especially in obesity.

In this study, we identify how epidermal γδ T cells participate in obesity-related skin complications such as wound healing and psoriasis-like inflammation. We find that CCR6 is expressed by a distinct subset of activated epidermal γδ T cells and CCL20 induces this subset to produce IL-17. Using previously published single-cell RNA sequencing (scRNAseq) data, we show that CCR6^+^ epidermal γδ T cells express IL-17–associated genes during psoriasis-like inflammation. We validate these findings in murine models of wound repair and imiquimod (IMQ)-induced psoriasis-like inflammation where CCR6 and/or IL17A are expressed by epidermal γδ T cells in vivo. Obesity increases the number of epidermal γδ T cells expressing CCR6 and IL-17A during wound healing, which underscores the significant impact of obesity on skewing toward an IL-17 proinflammatory response. Further, the identification of a subset of IL-17–producing, CCR6+ epidermal γδ T cells challenges the concept that all epidermal γδ T cells are programmed to function the same.

## Materials and methods

### Mice

C57BL/6N mice were purchased from Taconic Biosciences. Sixteen- to 20-wk-old male mice were studied for all cell culture and flow cytometry experiments. Male C57BL/6-Il17atm1Bcgen/J mice (JAX stock 018472) were purchased from the Jackson Laboratory. All C57BL/6-Il17atm1Bcgen/J mice used for experiments were purchased between the ages of 4 and 6 wk and used between the ages of 18 and 24 wk. Mice received access to food and water ad libitum and were housed in the animal facility at California State University San Marcos. For obesity studies, 6-wk-old male C57BL/6-Il17atm1Bcgen/J mice were fed either a high-fat diet (HFD) consisting of 60 kcal% fat diet (D12492; Research Diets) or normal chow diet (NCD) (D12450J; Research Diets) for 12 to 16 wk. All experimental procedures involving animals were reviewed and approved by the Institutional Animal Care and Use Committee of California State University San Marcos (21-003).

### Epidermal T cell culture

Epidermal cells were harvested from the back skin of 16- to 20-wk-old wild-type mice as previously described.^25^ Briefly, the back skin was removed, cut into 1-cm^2^ squares, and incubated on 0.3% trypsin-GNK (0.09% glucose, 0.84% sodium chloride, and 0.04% potassium chloride) at 37 °C with 5.0% CO_2_ for 3.5 h. The epidermis was then peeled from the dermis and shaken in 0.3% trypsin GNK with 0.1% DNase at 37 °C for 10 minutes. The cell solution was placed in Dulbecco’s Modified Eagle Medium with 10% heat inactivated fetal bovine serum, 2.5% HEPES buffer, 1% 100× nonessential amino acids, 1% 100 mM sodium pyruvate, 1% penicillin-streptomycin-glutamine, vitamins and 0.1% 2-mercaptoethanol. The cells were filtered through Sera-Separa filter columns (Evergreen Scientific), pelleted, and purified with Lympholyte M (Cedarlane Labs) prior to culture. The epidermal cells were plated in a 96-well plate containing RPMI 1640 media with 10% fetal bovine serum, 2.5% HEPES buffer, 1% nonessential amino acids, 1% sodium pyruvate, 1% penicillin-streptomycin-glutamine, 0.1% 2-mercaptoethanol, and 20 U/mL IL-2. A total of 2 μg/mL of concanavalin A (ConA) and 1 μg/mL of indomethacin were added at the initiation of cell culture. Twice a week fresh media without ConA and indomethacin was added. Cells were restimulated every 3 wk with 1 μg/mL of ConA and harvested when epidermal γδ T cells made up over 95% the cells in culture (typically 10 wk).

### Epidermal γδ T cell activation

For in vitro studies, 24-well plates were precoated with 1 μg/mL or 10 μg/mL of anti-CD3ε for 24 h at 37 °C with 5.0% CO_2_. For intracellular cytokine staining, the cells were stimulated for 6 h, and 5 µg/mL brefeldin A was added at hour 2. A fixation and permeabilization kit (BD Cytofix/Cytoperm) was used per the manufacturer’s instructions. The following antibodies were used: anti-CD3 (145-2C11), γδ TCR (GL3), CD25 (3C7), and CCR6 (29-2L17) (BioLegend). Flow cytometry was performed on an Accuri C6 (BD Biosciences) and data were analyzed with FlowJo software v10.10 (BD Biosciences).

### IMQ induction of psoriasis-like inflammation

A total of 5 mg of 5% IMQ cream (Fougera Pharmaceuticals) was applied to the right ear, and control cream (Vanicream; Pharmaceutical Specialties) was applied to the left ear of C57BL/6-Il17atm1Bcgen/J mice daily for 1 to 3 d.^6^ Mice were individually housed and monitored daily. At the end of the experiment, ears were harvested for staining and immunofluorescent microscopy.

### Wounding model

C57BL/6-Il17atm1Bcgen/J mice between 20 and 24 wk of age were anesthetized with a mixture of 1.75 L/m O_2_ and 2.5% of isoflurane and then received a 2-mm punch biopsy wound on 1 ear. Mice were individually housed and monitored daily. At 1 to 3 d postwounding, the ears were harvested for staining and immunofluorescent microscopy.

### Epidermal sheet immunofluorescent staining and microscopy

Epidermal sheets were prepared as previously described.^25^ Briefly, ears were split in half and placed on ammonium thiocyanate solution (1× dPBS, 3.6% ammonium thiocyanate) dermis side down, and incubated for 15 minutes, after which the epidermis was peeled from the dermis. Epidermal sheets were floated on 1× dPBS for 5 minutes, then floated on 2 μg/mL anti-Vγ5 and anti-CCR6 (BioLegend) and in some instances anti-CD69 or anti-CD127 (BioLegend) for 1 h at 37 °C with 5.0% CO_2_. Epidermal sheets were rinsed on 1× dPBS and mounted with Slowfade Gold Antifade Reagent with DAPI (Invitrogen). Slides were examined using an immunofluorescent microscope (Nikon DS-Qi2). Expression of IL-17A (GFP), CCR6 (PE), and the γδTCR (APC) were examined at the site of wounding or psoriasis induction. Images were captured using the Nikon NIS-Elements D 4.1300 64 program at original magnification ×200 and processed using Adobe Photoshop 2022. Total epidermal γδ T cell number as well as the number of epidermal γδ T cells that expressed CCR6 and/or IL-17 were quantified and then calculated as cells/mm^2^. In both the psoriasis and wounding experiments, a total of 7 regions were photographed per epidermal sheet per ear, accounting for both wounded and nonwounded ears, as well as IMQ-treated and untreated control ears. Each ear provided 2 epidermal sheets. In total, 672 images were captured, analyzed, and quantified between the IMQ and wounding models.

### scRNAseq data processing and analysis

Publicly available scRNAseq data were downloaded as FASTQ files from the National Institutes of Health Gene Expression Omnibus database (GSE149121).^19^ Files were downloaded to the Linux terminal and transferred to the Cell Ranger Analysis Pipeline terminal (10x Genomics). The study by Liu et al.^19^ used single-cell transcriptomics of CD45^+^ cells from mice treated with IMQ for 7 d. All fastq files were individually run through Cell Ranger Analysis Pipeline v6.1 to perform gene quantification and sequence alignment to the 10x Genomics mouse reference genome (mm10). Additionally, Cell Ranger was utilized to subsample experiment reads and produce an aggregated gene expression matrix. Once aggregation and structuring of the data was completed, Cell Ranger generated a cloupe file that was uploaded to the visualization software Loupe Browser v6.0 (10x Genomics) to be used for downstream analysis of scRNAseq data. scRNAseq data were then uploaded to Loupe Browser for further analysis. Epidermal γδ T cell populations were identified by their expression of *Tcrg-v5*, *Fcer1g*, and *CD3.* Any contaminating non epidermal γδ T cell populations were eliminated based on their positive expression of *Cd4*, *Cd8*, *Tcrg-v4*, *Tcrg-v6*, *Krt5*, *Krt10*, *Cd207*, or *Lyz1*. Once the epidermal γδ T cell clusters were identified, further analysis was performed by clustering the epidermal γδ T cells into subsets (CCR6^+^IMQ, CCR6^+^control, CCR6^–^IMQ, CCR6^–^control). Subsets were compared between IMQ-treated and untreated control mice and differentially expressed genes (DEGs) were exported from Loupe Browser and submitted to Ingenuity Pathway Analysis (IPA) (Qiagen) for core analysis.

### Statistical analysis

Unpaired Student *t* tests and *z* test were used to analyze T cell numbers and corresponding cytokine production. All statistical tests performed using GraphPad Prism software version 9.5.1 (GraphPad Software). All findings are considered significant at *P *<* *0.05. Log2-fold change in Loupe Browser was calculated by using the localized ratio of normalized mean gene unique molecular identifier counts in each cluster relative to all other clusters.

## Results

### CCR6 was upregulated by a subset of epidermal γδ T cells upon anti-CD3 stimulation

**Figure 1.**
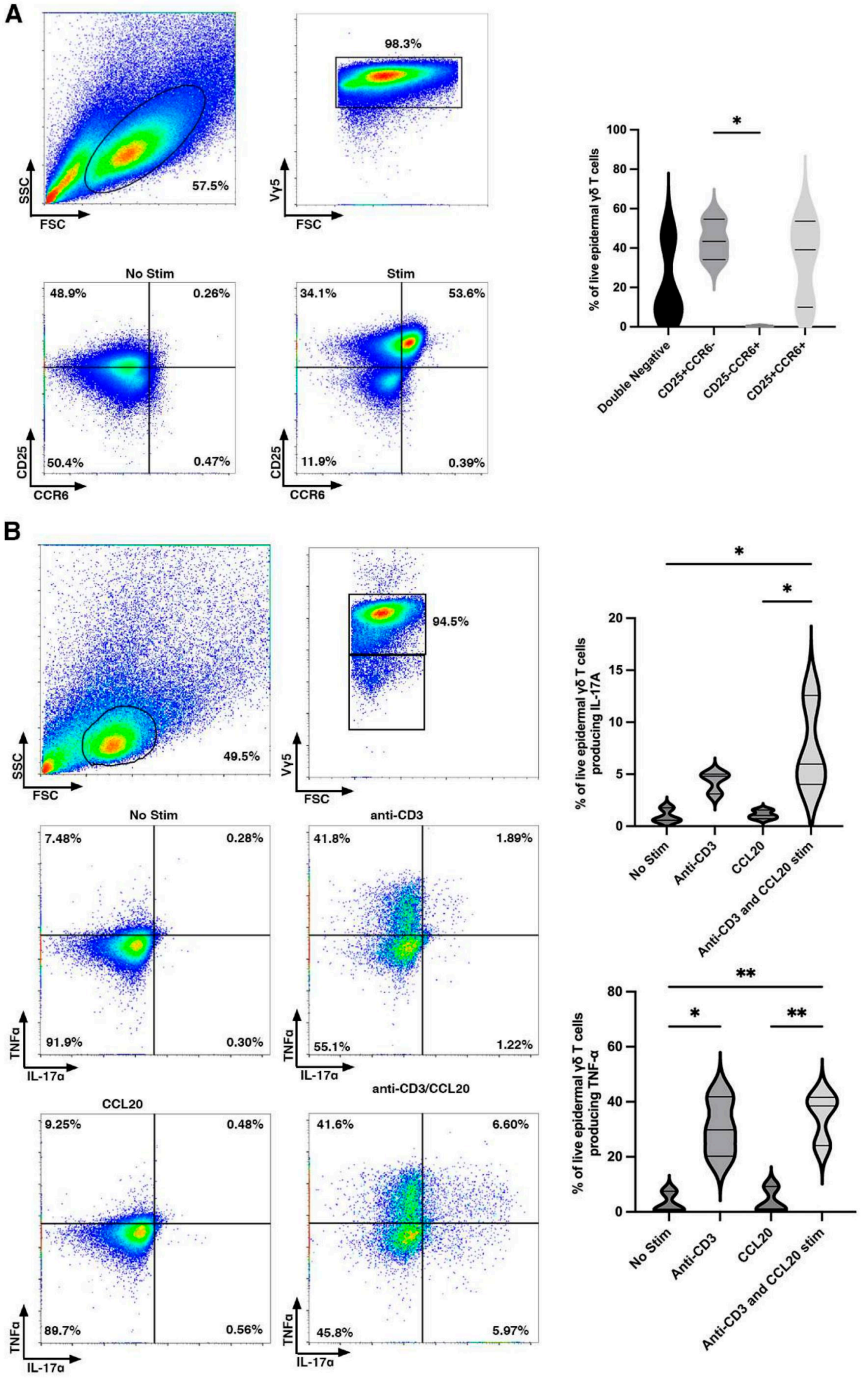
CCR6 is upregulated by a subset of CD25^+^ epidermal γδ T cells upon anti-CD3 stimulation. (A) Flow cytometric analysis of epidermal cells isolated from B6 mice, cultured for 9 to 15 wk (>95% epidermal γδ T cells), and stimulated with anti-CD3. Live, γδ TCR^+^ cells are gated and CCR6 and CD25 analyzed (n = 3). (B) Flow cytometric analysis of epidermal Vγ5 T cells stimulated in the presence or absence of anti-CD3 and/or CCL20 for 6 h. Live, Vγ5^+^ T cells are gated and TNF-α and IL-17A analyzed (n = 3). Data represent the mean ± SD. *P < 0.05; **P < 0.01. FSC, forward scatter; SSC, side scatter.

Epidermal γδ T cells do not express CCR6 on the cell surface during homeostatic conditions.^24^ While resting epidermal γδ T cells do not express CCR6, we tested whether CCR6 is expressed upon activation. Epidermal γδ T cell lines were stimulated with or without anti-CD3 stimulation for 24 h and CCR6 expression was examined by flow cytometry. Live, Vγ5 TCR^+^ cells were gated and analyzed for CCR6 expression. Upon stimulation with anti-CD3 for 24 h, a subset of epidermal γδ T cells expressed CCR6 and CD25 (Fig. 1A). Of note is the role that may be played by IL-2 in the CCR6-expressing population.

### CCL20 increased IL-17A but not TNF-α production by activated epidermal γδ T cells

Previous studies showed that CCR6^+^ dermal Vγ4 T cells secrete IL-17A in response to skin damage and during psoriasis.^10^^,^^13^^,^^26^ While a small number of epidermal γδ T cells produce IL-17 during wound repair and contact hypersensitivity, it is unknown if IL-17 production is regulated by CCL20 and whether this is a distinct CCR6^+^ population.^12^^,^^13^ To determine whether IL-17A and TNF-α production by epidermal γδ T cells is augmented by the CCR6 ligand, CCL20, we examined epidermal γδ T cells poststimulation with CCL20 and/or anti-CD3. There was not a significant increase in epidermal γδ T cells producing IL-17A poststimulation with anti-CD3, but there was a significant increase in epidermal γδ T cells producing TNF-α (Fig. 1B). CCL20 alone did not increase the percent of epidermal γδ T cells producing either IL-17A or TNF-α. Furthermore, CCL20 administered with anti-CD3 stimulation did not induce more epidermal γδ T cells to produce TNF-α than anti-CD3 alone (Fig. 1B). However, CCL20 administered with anti-CD3 stimulation significantly increased the percent of epidermal γδ T cells producing IL-17A. Interestingly, upon the addition of anti-CD3 and CCL20, there were 4 functional subsets of epidermal γδ T cells: TNF-α^–^/IL-17A^–^, TNF-α^+^/IL-17A^–^, TNF-α^–^/IL-17A^+^, and TNF-α^+^/IL-17A^+^. This challenges previous studies suggesting that epidermal γδ T cells are preprogrammed away from a Tγδ17 fate and that they all have the same function.

### Epidermal γδ T cells expressing CCR6 exhibited a unique gene expression profile

**Figure 2.**
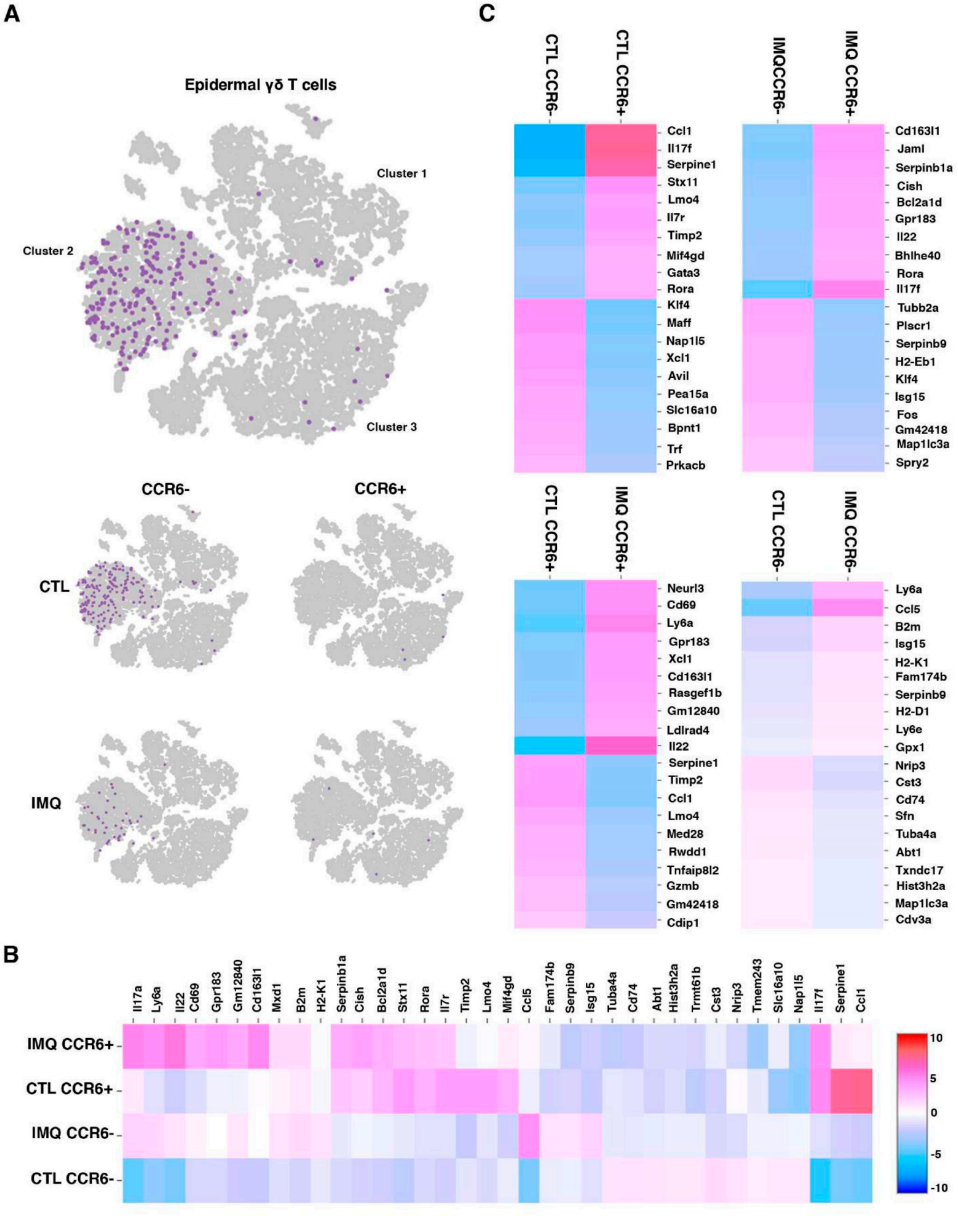
CCR6^+^ epidermal γδ T cells exhibit a Tγδ17 gene expression profile. Using publicly available scRNAseq data,^20^ the total skin cell population (n=18,040) was filtered to epidermal γδ T cells (n = 236). (A) Uniform Manifold Approximation and Projection plot showing that epidermal γδ T cells can be identified in 3 distinct clusters. Based on treatment group and CCR6 expression, epidermal γδ T cells were clustered further into CCR6^+^IMQ, CCR6^+^control, CCR6^–^IMQ, and CCR6^–^control groups. In the treatment group, the ratio of CCR6^+^ to CCR6^–^ cells increased from 1 to 48 in the control group to 1 to 9. (B) Differential gene expression between 4 different epidermal γδ T cell populations within treatment groups displayed in a heatmap. (C) Dual heatmap rendering of differential gene expression between specific epidermal γδ T cell groups.

To examine gene expression by CCR6^+^ epidermal γδ T cells during psoriasis, publicly available scRNAseq data from Liu et al.^19^ were reanalyzed with a focus on CCR6^+^ or CCR6^-^ epidermal γδ T cells. In this study, RNA was isolated from C57BL/6J mouse skin treated with or without 5% Imiquimod for 7 d and scRNAseq was performed. Liu et al. reported a cluster of epidermal γδ T cells in a t-distributed stochastic neighbor embedding plot indicating a broad population with a potential for subsets of epidermal γδ T cells with differing gene expression. To specifically analyze epidermal γδ T cells, target cells were sorted from the overall cell count using Loupe Browser based on well-established marker genes (*Cd3^+^*, *Tcrg-v5^+^*, *Trdc^+^*, *Cd4^–^*, *Cd8^–^*). Minor populations of contaminant keratinocytes and Langerhans cells were excluded by removing cells exhibiting high levels of *Krt5*, *Krt10*, and *Krt14* for the keratinocytes and high levels of *Cd207* for the Langerhans cells, yielding a total of 236 epidermal γδ T cells for analysis (Fig. 2A). These exclusions produced 3 distinct epidermal γδ T cell populations (labeled clusters 1–3 in Fig. 2A).

Epidermal γδ T cells were further clustered based on treatment group and CCR6 expression (CCR6^+^IMQ, CCR6^+^control, CCR6^–^IMQ, CCR6^–^control) (Fig. 2A). Prior to IMQ treatment, CCR6^–^ epidermal γδ T cells were represented in clusters 1, 2, and 3. However, post–IMQ treatment, the CCR6^–^ epidermal γδ T cells predominantly centralized to cluster 2. Pre–IMQ treatment, CCR6^+^ epidermal γδ T cells were represented in cluster 3, while post-treatment the CCR6^+^ epidermal γδ T cells were represented in both clusters 2 and 3. A locally distinguishing differential gene expression analysis was run between the CCR6^+^ and CCR6^–^ epidermal γδ T cell populations with and without IMQ treatment and a heatmap was generated showcasing the top 10 significantly upregulated genes per cluster based on the logarithmic fold change of gene expression between each group during paired comparison (Fig. 2B).

Interestingly, both CCR6^+^ subsets differentially express the transcription factor *Rora*, which regulates both CCR6 and IL17 expression (Fig. 2B).^27^ To better characterize each individual epidermal γδ T cell population, we analyzed DEGs between paired subsets. Comparing the CCR6^+^control and CCR6^–^control groups, we observed that the CCR6^+^control gene profile was more inflammatory, favoring immune cell infiltration (*Ccl1*, *Serpine1*, *Il17f*), whereas the CCR6^–^control group did not exhibit the upregulation of any DEGs above 5-fold. However, when examining gene expression changes exceeding 4-fold, the CCR6^–^control subset demonstrated increased expression of genes related to mitotic cell cycle, DNA binding, and cell motility (*Maff*, *Klf4*) (Fig. 2C, top left). Next, in the comparison between CCR6^+^IMQ and CCR6^–^IMQ treatment groups, the CCR6^+^IMQ subset exhibited a Tγδ17 inflammatory profile, with a >5-fold upregulation of *Il17f*, along with lower-level increases in *Rora*, *IL22*, and *Jaml*. The CCR6^–^IMQ subset experienced a >3-fold upregulation in genes associated with cytokinesis, proliferation, and cell motility (*Tubb2a*, *Fos*, *Plscr1*) (Fig. 2C, top right).

In the comparison among CCR6^+^ groups, the CCR6^+^IMQ subset consistently exhibited a proinflammatory profile compared with all other subsets, with a >5-fold change in *Il22* expression (Fig. 2C, bottom left). On the other hand, the CCR6^+^control subset still showed a >3-fold upregulation in genes associated with immune cell infiltration (*Ccl1*, *Serpine1*). Last, the comparison among CCR6^–^ subsets indicated minimal gene upregulation except for potential immune cell recruitment (*Ccl5*) in the CCR6^–^IMQ subset (Fig. 2C, bottom right). Our results suggest that CCR6-expressing epidermal γδ T cells in psoriasis exhibit a skewed IL-17–focused response.

### Psoriasis increased MYC pathway signaling by CCR6^+^ epidermal γδ T cells

**Figure 3.**
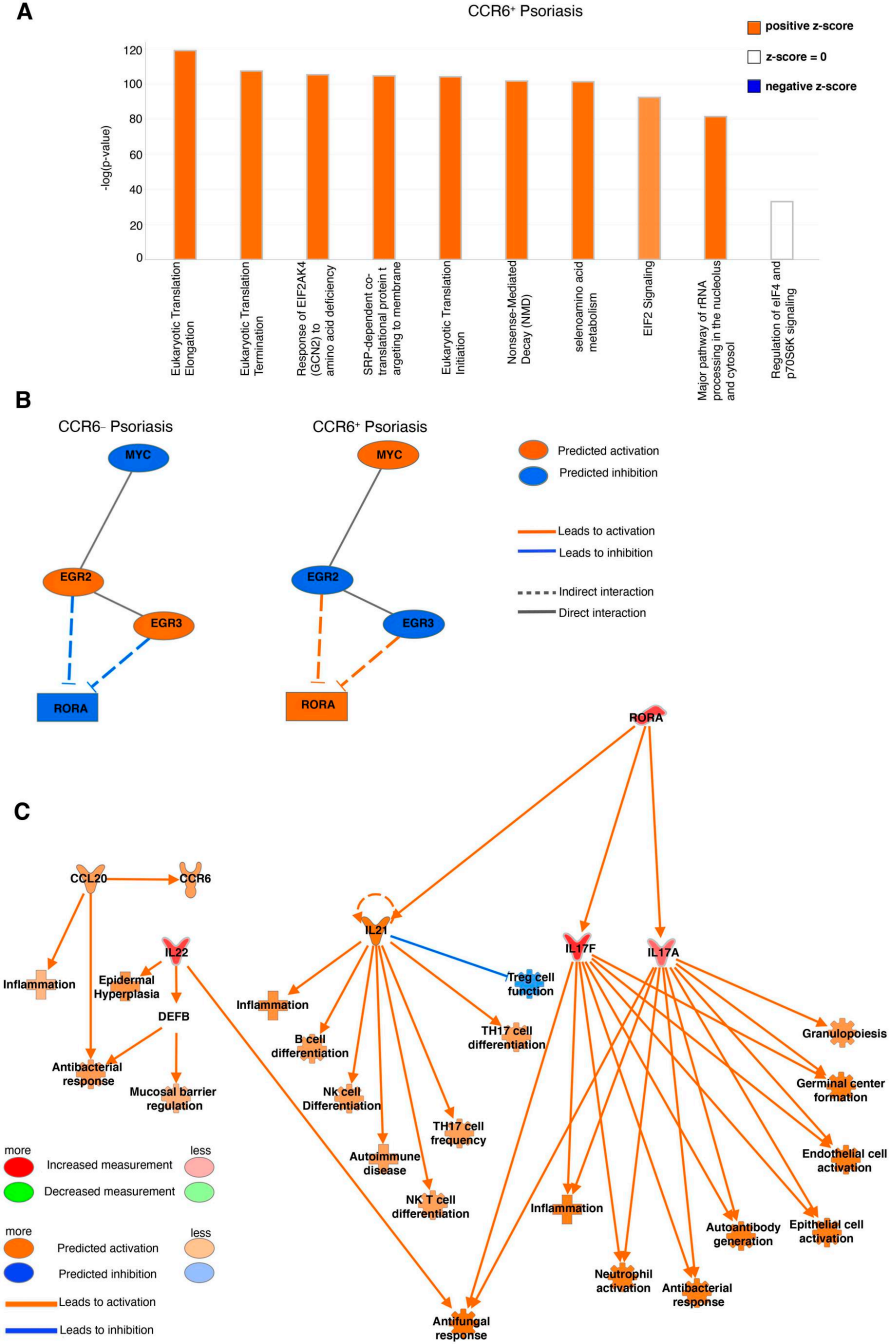
Canonical pathway analysis of differentially expressed genes from CCR6^+^ and CCR6^-^ epidermal γδ T cells during IMQ-induced psoriasis identify Myc pathway. (A) Top differentially expressed genes between CCR6^+^IMQ and CCR6^–^IMQ from publicly available scRNAseq data^20^ were clustered into canonical pathways using IPA Knowledge Base platform. A positive *z* score shows pathway activation. A negative *z* score shows the pathway is inhibited. No *z* score shows the pathway is neither activated nor inhibited. Bars are arranged by statistical significance. (B) Network analysis identifies upstream regulator Myc (orange indicating activation), with IPA Knowledge Base predicting a linked activation with Rora. (C) Network downstream analysis of Rora pathway with orange predicting activation, blue predicting inhibition, and red predicting an increased measurement between subsets (CCR6^+^IMQ vs. CCR6^-^IMQ epidermal γδ T cells).

IPA core analysis was performed to further elucidate the biological processes and molecular mechanisms that differentiate CCR6^+^ epidermal γδ T cell subsets in psoriasis. Top DEGs were clustered into canonical pathways using the IPA Knowledge Base platform (Fig. 3A). Comparison of CCR6^+^IMQ with CCR6^–^IMQ subsets revealed eukaryotic translation elongation, eukaryotic translation termination, response of EIF2AK4 (GCN2) to amino acid deficiency, SRP-dependent cotranslational protein targeting to membrane, eukaryotic translation initiation, nonsense-mediated decay, selenoamino acid metabolism, EIF2 signaling, and major pathway of rRNA processing in the nucleolus and cytosol as the significantly upregulated pathways of CCR6^+^IMQ subsets in psoriasis when compared with CCR6^–^IMQ subsets in psoriasis (Fig. 3A). Examination of significantly differentiated upstream regulators between these subsets revealed that the Myc pathway was significantly upregulated within the population of CCR6^+^IMQ epidermal γδ T cells when compared with CCR6^–^IMQ epidermal γδ T cells (Fig. 3B). The Myc pathway was predicted by the IPA Knowledge Base to be linked with activation of the transcription factor *Rora* (Fig. 3B). Analysis of the *Rora* downstream pathway showed that upregulation of *Rora* by CCR6^+^IMQ epidermal γδ T cells directly activates the *Il17a* and *Il17f* expression found within CCR6^+^IMQ epidermal γδ T cell subsets. Furthermore, this downstream pathway activation by *Rora* was predicted by IPA Knowledge Base to lead to activation of *Il22* as well as of the CCL20/CCR6 axis in CCR6^+^IMQ epidermal γδ T cell subsets (Fig. 3C). Our results suggest that the activation of the Myc pathway within CCR6^+^IMQ epidermal γδ T cell subsets contributes to the increased Tγδ17 cytokine profile observed within CCR6^+^IMQ epidermal γδ T cell subsets.

### CCR6 was upregulated within 2 d post–IMQ treatment

**Figure 4.**
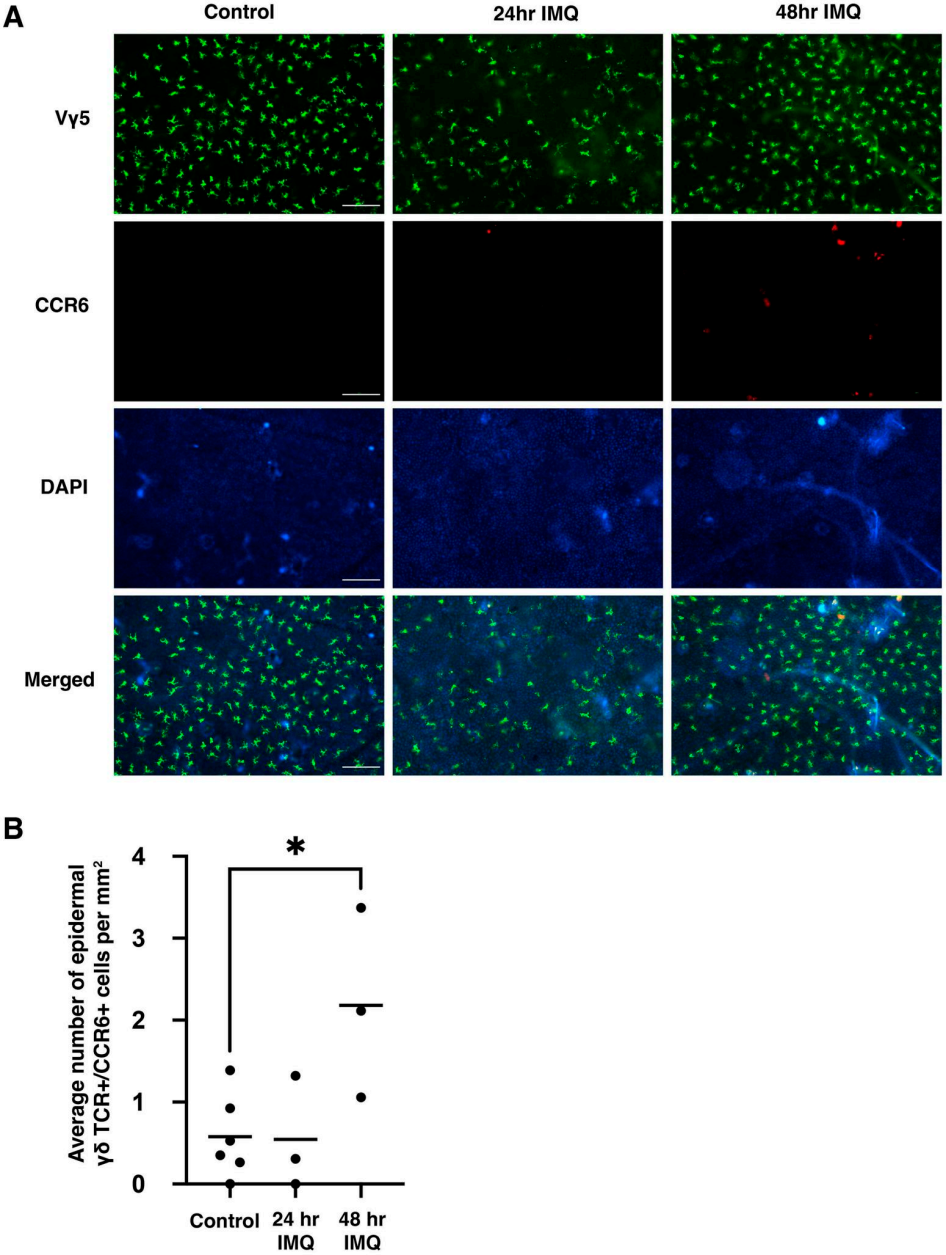
CCR6 is upregulated within the first 48 h post–IMQ treatment. (A) Representative immunofluorescent images of epidermal sheets from B6 mice at various time points post–IMQ treatment. Scale bars = 100 μm. (B) Quantification of CCR6^+^ epidermal γδ T cells at different time points post–IMQ treatment (n = 3–6 mice/group). A minimum of 7 fields of view for each mouse were used for analysis and then averaged to form 1 data point. *P < 0.05.

Epidermal γδ T cells act very early in skin immunity, suggesting that they play roles prior to dermal γδ T cells and other key populations in psoriasis. To determine when CCR6 is upregulated by epidermal γδ T cells in response to IMQ treatment, a time course was performed. During wound healing, epidermal γδ T cells become activated and produce growth factors and cytokines within the first 2 d postwounding. Thus, we examined CCR6 expression on epidermal γδ T cells at days 0, 1, and 2 post–IMQ application. This time point is earlier than typically examined and prior to psoriasis-like symptoms and inflammation. As expected, there is little to no CCR6 expression by epidermal γδ T cells in nontreated control skin (Fig. 4A and B). However, the number of CCR6-expressing cells increased significantly 2 d post-treatment (Fig. 4A and B). Overall, these data indicates that CCR6 expression by epidermal γδ T cells is upregulated early upon IMQ treatment prior to the infiltration of dermal γδ T cells. CD69 and CD127 were also identified in the scRNAseq analysis as upregulated by CCR6^+^ epidermal γδ T cells, so we also validated CD69 and CD127 expression. At 48 h post–IMQ treatment, over 90% of CCR6^+^ epidermal γδ T cells analyzed expressed CD69/CD127 (Fig. S1).

### Psoriasis increased IL-17 and CCR6 production by epidermal γδ T cells

**Figure 5.**
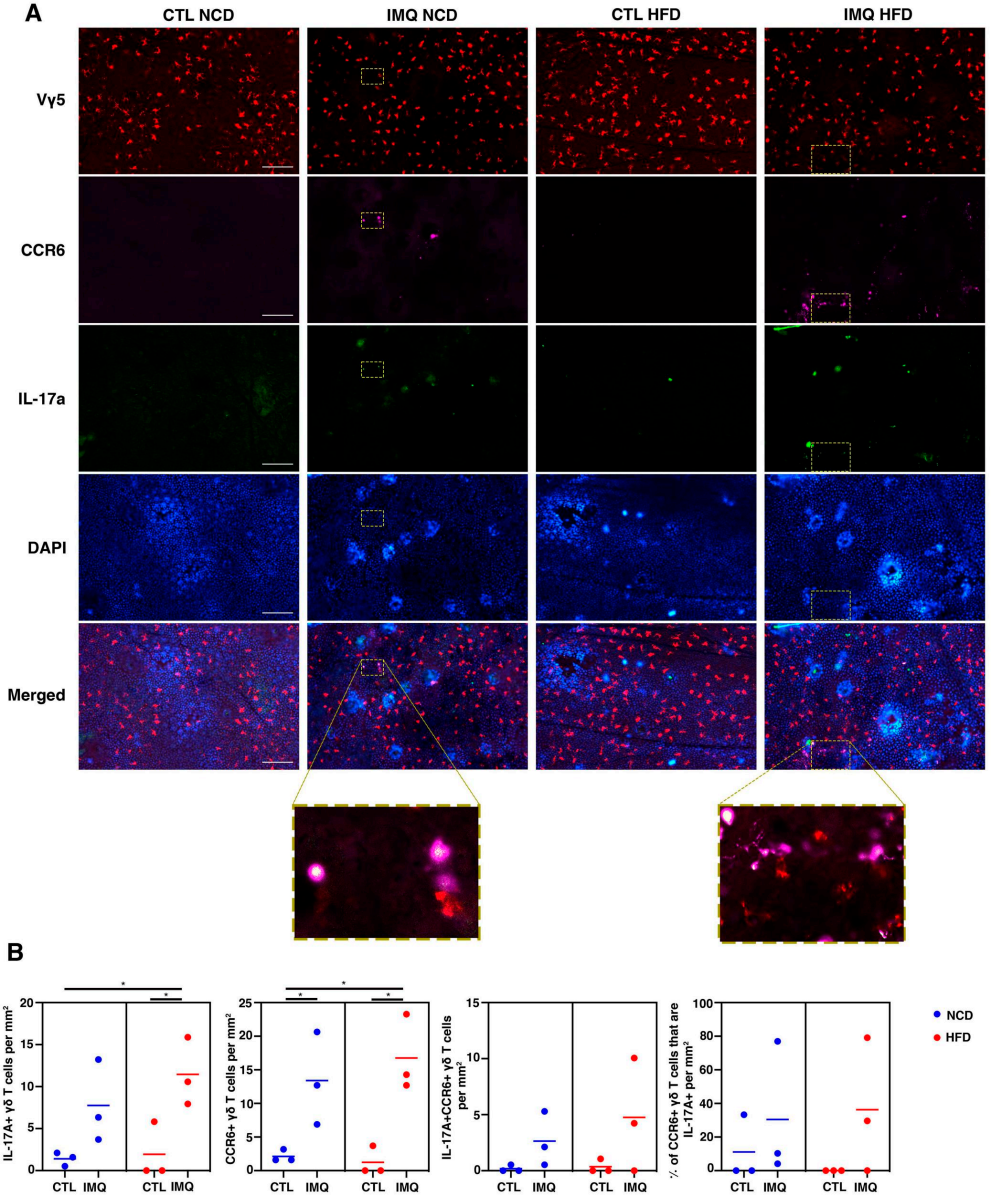
IMQ-induced psoriasis increases IL-17A and CCR6 expression by epidermal γδ T cells, while obesity does not further increase expression. (A) Representative immunofluorescent images of epidermal sheets from C57BL/6-Il17atm1Bcgen/J mice treated with and without IMQ for 2 d. Scale bars = 100 μm. (B) Quantification of IL-17A^+^, CCR6^+^, and IL-17A^+^CCR6^+^ epidermal γδ T cells with and without IMQ treatment (n = 3 mice/group). A minimum of 7 fields of view for each mouse were used for analysis and then averaged to form 1 data point. *P < 0.05. CTL, control.

To validate the scRNAseq findings that CCR6^+^ epidermal γδ T cells produce IL-17A during psoriasis, we examined IL-17A production in vivo using IL-17A GFP reporter mice. To establish when CCR6 upregulation occurs post–IMQ treatment, C57BL/6J wild-type mice were treated with IMQ for 0 to 2 d. CCR6 expression by epidermal γδ T cells peaked at 2 d post–IMQ treatment (Fig. 4). Thus, in our studies, mice received IMQ treatment for 2 d prior to analysis. This time point is similar to previous studies in wound healing models when epidermal γδ T cell activation was observed within 6 h of wounding and function persisted for at least 2 d.^16^^,^^28^ Epidermal sheets were costained for Vγ5, and CCR6, while IL-17A was detected with GFP, and epidermal γδ T cells were quantified per mm^2^ (Fig. 5A). During IMQ-induced psoriasis, there were more IL-17A–producing epidermal γδ T cells than controls (Fig. 5A and B), but this increase was just short of reaching significance (*P *= 0.075) (Fig. 5B). Similarly, there were more CCR6-expressing epidermal γδ T cells upon IMQ treatment (Fig. 5A and B). While CCR6^+^IL-17A^+^ epidermal γδ T cells were easily identified in IMQ-treated mice, the increases did not reach significance. Similarly, no significant differences were found in the percentage of CCR6^+^ epidermal γδ T cells that were also IL-17A^+^ (Fig. 5A and B). However, given that the epidermal γδ T cells express both CCR6 and IL17A in a time-dependent manner, CCR6 may be upregulated prior to IL-17A.

### IL-17 and CCR6 upregulation by epidermal γδ T cells in obese and lean mice was similar in psoriasis

To determine whether there is an impact of obesity on CCR6 expression or IL-17 production by epidermal γδ T cells in psoriasis-like inflammation, IL-17A GFP reporter mice were fed either an NCD or HFD for 12 to 16 wk and then received IMQ treatment for 2 d. There was a significant increase in IL-17 production by epidermal γδ T cells within the IMQ group when compared with the control group in mice fed an HFD (Fig. 5A and B). This was now significant but was only reaching significance in the lean control group, suggesting a subtle effect of obesity on IL-17 production by epidermal γδ T cells. Upregulation of CCR6 by epidermal γδ T cells in psoriasis occurs in both NCD- and HFD-fed mice to a similar degree, suggesting that obesity does not exacerbate CCR6 expression at this time point of IMQ-induced psoriasis onset (Fig. 5A and B).

### CCR6 was upregulated within 1 d and downregulated by 3 d postwounding

**Figure 6.**
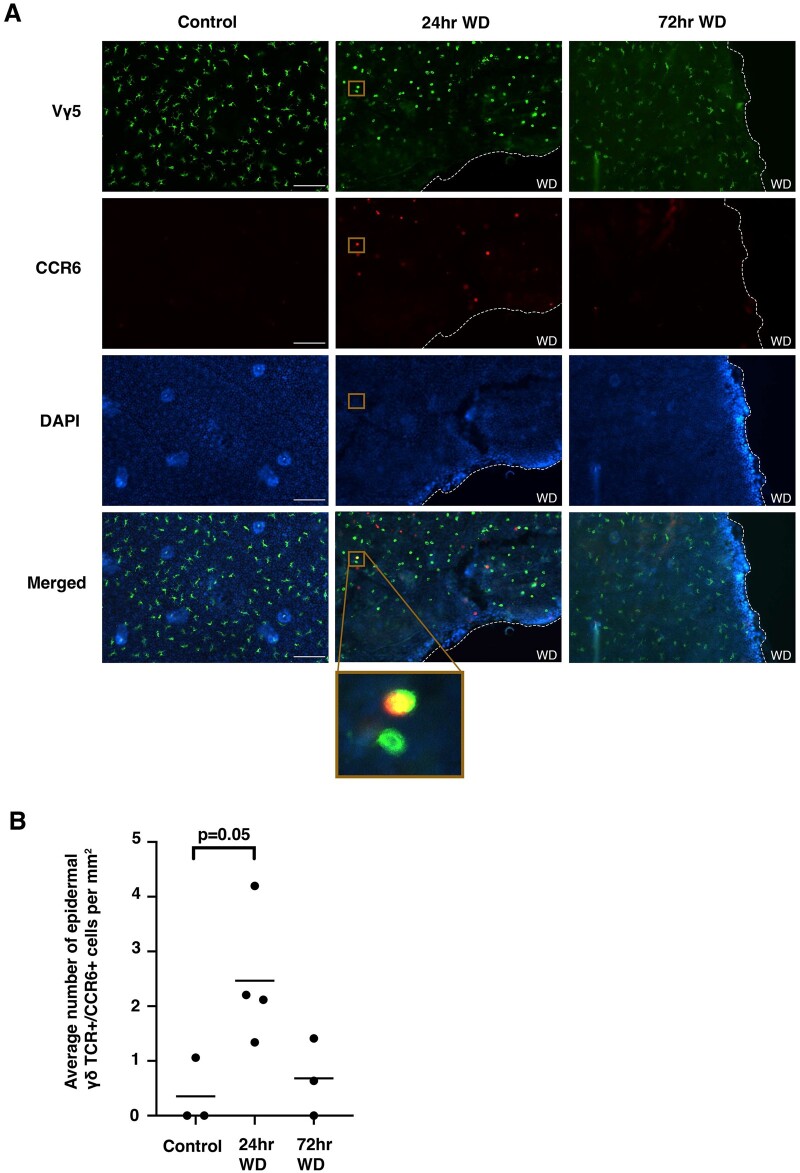
CCR6 was upregulated within the first 24 h postwounding and downregulated by 72 h postwounding. (A) Representative immunofluorescent images of epidermal sheets from B6 mice at various time points postwounding. The wound site is indicated with a dotted line. Scale bars = 100 μm. (B) Quantification of CCR6^+^ epidermal γδ T cells at different time points postwounding (n = 3–4 mice/group). A minimum of 7 fields of view for each mouse were used for analysis and then averaged to form 1 data point. WD, (Wound).

To determine how CCR6 is regulated by epidermal γδ T cells in response to wounding, we performed a time course. Epidermal γδ T cells became activated and produced growth factors and cytokines for the first 2 d postwounding. Thus, we examined CCR6 expression on epidermal γδ T cells at days 0, 1, 2, and 3 postwounding. As expected, there was little to no CCR6 expression by epidermal γδ T cells in nonwounded control skin (Fig. 6A and B). However, the number of CCR6-expressing cells increased significantly 1 d postwounding, with CCR6 expression returning to nonwounded control levels 3 d postwounding (Fig. 6A and B). Overall, these data indicate that CCR6 expression by epidermal γδ T cells is early and temporal during wound repair instead of being constitutive as in dermal γδ T cells.

### IL-17A–expressing epidermal γδ T cells were significantly increased at the wound site

**Figure 7.**
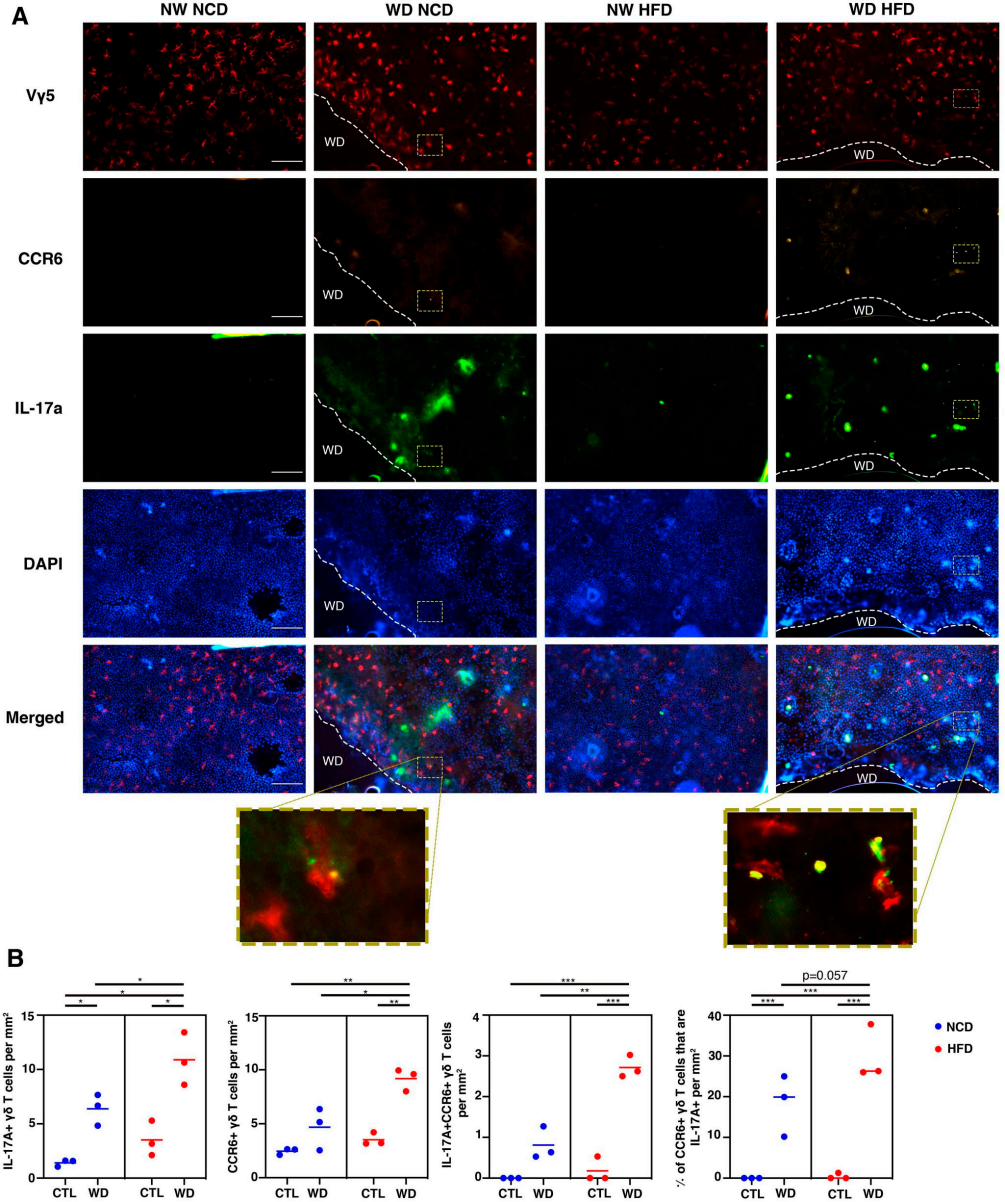
IL-17A and CCR6 expression by epidermal γδ T cells was significantly elevated in wounded mice, while obesity increased IL-17A production and CCR6 expression by epidermal γδ T cells at the wound site. (A) Representative immunofluorescent images of epidermal sheets from C57BL/6-Il17atm1Bcgen/J mice 1 d postwounding. The wound site is indicated with a dotted line. Scale bars = 100 μm. (B) Quantification of IL-17A^+^, CCR6^+^, and IL-17A^+^CCR6^+^ epidermal γδ T cells with and without wounding (n = 3 mice/group). A minimum of 7 fields of view for each mouse were used for analysis and then averaged to form 1 data point. *P < 0.05; **P < 0.01; ***P < 0.001. CTL, control.

CCR6 and IL-17 production by epidermal γδ T cells were examined in wounded and nonwounded IL-17A reporter mice. IL-17A–producing epidermal γδ T cells were increased in wounded mice as compared with their nonwounded counterparts. CCR6-expressing epidermal γδ T cells from wounded NCD mice were increased in 2 of the 3 wounded mice as compared with nonwounded mice (Fig. 7A and B). A total of 20% of the CCR6^+^ epidermal γδ T cells expressed IL-17A in wounded mice compared with 0% in nonwounded mice. Together, these data suggest that wounding induces epidermal γδ T cells to upregulate IL-17, and this occurs on a proportion of CCR6^+^ cells.

### Obesity increased IL-17A production and CCR6 expression by epidermal γδ T cells at the wound site

Previous studies have shown that obesity alters epidermal γδ T cell number and function during wound repair.^5^^,^^11^ To determine if there is a shift in epidermal γδ T cell function toward a Tγδ17 phenotype in obesity IL-17A GFP reporter mice were fed either an NCD or an HFD for 12 to 16 wk and then were wounded for 1 d prior to analysis. Obese mice exhibited an increase in IL-17A–producing epidermal γδ T cells in wounded vs nonwounded mice (Fig. 7B). In addition, at the wound site, obese mice exhibited significantly elevated numbers of IL-17A–producing epidermal γδ T cells as compared with their NCD counterparts (Fig. 7B). CCR6 expression by epidermal γδ T cells at the wound site was also significantly increased in obese mice as compared with lean mice (Fig. 7A and B). The number of CCR6^+^ epidermal γδ T cells that simultaneously expressed IL-17A is also higher in obese mice upon wounding and as compared with lean wounded mice (Fig. 7B). There was also an increase in the percentage of CCR6^+^ epidermal γδ T cells concurrently expressing IL-17A, which was nearly significant in the wounded obese group as compared with the wounded lean group (*P *= 0.057) (Fig. 7B). Together, these data show that obesity increases Tγδ17 epidermal γδ T cells.

## Discussion

Epidermal γδ T cells exhibit a variety of functional responses, including Tγδ1, Tγδ2, and Tγδ17, for roles in wound repair, tumor cytolysis, and contact hypersensitivity.^12^^,^^16^^,^^29^ These functions are regulated via costimulation through receptors such as JAML and CD100 in wound repair and cytokine reception such as IL-1β in contact hypersensitivity.^13^^,^^30^ Furthermore, it is important to note that epidermal γδ T cells express a canonical Vγ5Vδ1 TCR and exist as a functionally separate population from dermal γδ T cells, which express either the Vγ4 or Vγ6 TCR. In fact, the role that IL-23 plays in the IL-17 response differs between epidermal and dermal γδ T cells in the context of skin inflammation.^10^^,^^12^ IL-23 induces IL-17 production within dermal γδ T cell populations, while IL-23 stimulation does not induce IL-17 production of epidermal γδ T cells, as they lack the IL-23 receptor.^10^^,^^12^ Because epidermal γδ T cells express a unique and separate γδ T cell population from those found in the dermis, it has been postulated that the entirety of the epidermal γδ T cell population must serve the same functions within the epidermis.^12^^,^^30^ Here, we suggest that within this unique population of γδ T cells, there are in fact functional subsets with skewed abilities, and these subsets can be increased by environmental factors such as obesity. Previously, epidermal γδ T cells with IFN-γ– and IL-17–secreting abilities were identified, but there are currently no markers to further define these cells and to define whether these are specific subsets or they exhibit cellular plasticity.^13^ We show that the expression of CCR6, along with CD69 and CD127, upon activation defines a subset of epidermal γδ T cells with Tγδ17 functional capabilities. Thus, chemokines such as CCL20 can direct the function of a distinct epidermal γδ T cell subset during activation.

It is possible that identifying subsets of epidermal γδ T cells has been difficult because the markers are more easily observed during activation. Particularly for the CCR6^+^ subset, T cell activation is required for the upregulation of CCR6 and then chemokine reception is required for IL-17A production. Our data suggest that there is a temporal requirement for TCR signaling, followed by CCR6 upregulation and CCL20 reception to get IL-17A production. We reveal an association between the Tγδ17 profile observed in CCR6^+^ epidermal γδ T cells during psoriasis and the upstream transcription factor Myc. Myc is an early response gene in T cell activation.^31^ Expression of Myc is regulated by TCR signal strength and cytokine reception such as IL-2.^32^^,^^33^ This correlates well with our finding that CCR6^+^ epidermal γδ T cells also express CD25. Further, we find that Myc activation is positively associated with RORα, primarily attributed to the inhibition of early growth response protein 2 (EGR2). EGR2 deficiency in CD4 T cells leads to an increase in IL-17 expression.^32^ Furthermore, it has been established that the overexpression of Myc by γδ natural killer T cells results in EGR2 deficiency.^33^ Our results are consistent with published studies that indicate that RORα directly regulates the expression of both CCR6 and IL-17.^34^ When considering the known interaction between RORα, EGR2, and Myc, our results suggest that the pathway involving Myc, EGR2, and RORα may serve as a promising focus for understanding the underlying mechanisms behind the expression of the Tγδ17 profile in CCR6^+^ epidermal γδ T cells during skin inflammation.

Obesity increases the number of CCR6- and IL-17–expressing epidermal γδ T cells during the early stages of wound repair but not during IMQ-induced psoriasis. It is possible that there is a set number of epidermal γδ T cells with preprogrammed Tγδ17 function and that IMQ-induced psoriasis activates the entire subset. Thus, obesity would not induce an additional increase, while wounding only induces some of the Tγδ17 epidermal T cells and obesity further increases the number of activated Tγδ17 epidermal T cells. Previous research has demonstrated that obesity not only upregulates IL-17, but also boosts the production of CCL20.^23^^,^^35^ This dysregulation of the epidermis corresponds to our previous findings that show the cellular composition and organization of the epidermis is altered in HFD and db/db mouse models of obesity due to hyperglycemia and chronic inflammation. In diabetes and obesity, T cell and keratinocyte numbers and tissue repair functions are compromised. Epidermal γδ T cell dysfunction plays a role in the reduced number and increased differentiation of keratinocytes in both models.^5^^,^^11^ Another theory is that obesity does not increase IL-17– and CCR6-expressing epidermal γδ T cells during psoriasis because of the experimental timeline we used. During IMQ-induced psoriasis, IL-17 production is evident in skin resident T cell populations, and continues to rise throughout the 7 d IMQ treatment.^19^ For this study we chose the time point in which we observed the most CCR6^+^ epidermal γδ T cells in lean mice, but obesity may alter that time point.

CCR6 has been associated with numerous diseases including psoriasis and is a target for therapeutic intervention, but CCR6 targeting drugs have not yet been approved by the Food and Drug Administration.^36^^,^^37^ Cells including dermal γδ T cells utilize CCR6 to traffic to the epidermis during inflammation.^20^^,^^38^ Thus, drugs that block the function of CCR6 or interaction with CCL20 would reduce the recruitment of IL-17–producing T cells. Epidermal γδ T cells normally reside in the basal layer between keratinocytes and are not known for migrating far within or outside of the epidermis.^16^^,^^39^ Here, we have identified a clear subset of epidermal γδ T cells that upregulate CCR6 and thus would also be targeted with CCR6-specific therapeutics. It is now clear that CCR6^+^ epidermal γδ T cells contribute to Tγδ17-associated responses in psoriasis and wound healing, challenging previous assumptions that other dermal and infiltrating cell types were the only IL-17 producers.^10^^,^^24^ Further, these data showcases the impact of obesity on epidermal γδ T cell subsets and function in inflammatory settings.

## Supplementary Material

vkae011_Supplementary_Data

## Data Availability

Publicly available scRNAseq data were downloaded as FASTQ files from the National Institutes of Health Gene Expression Omnibus database (GSE149121).

## References

[1] Abramczyk R , Queller JN, Rachfal AW, Schwartz SS. Diabetes and psoriasis: different sides of the same prism. Diabetes Metab Syndr Obes Targets Ther. 2020;13:3571–3577. 10.2147/DMSO.S273147 PMC7548229 33116708

[2] Sen CK. Human wound and its burden: updated 2020 compendium of estimates. Adv Wound Care (New Rochelle). 2021;10:281–292. 33733885 10.1089/wound.2021.0026 PMC8024242

[3] Huangfu L , Li R, Huang Y, Wang S. The IL-17 family in diseases: from bench to bedside. Signal Transduct Target Ther. 2023;8:1–22. 37816755 10.1038/s41392-023-01620-3 PMC10564932

[4] McGeachy MJ , Cua DJ, Gaffen SL. The IL-17 family of cytokines in health and disease. Immunity. 2019;50:892–906. 30995505 10.1016/j.immuni.2019.03.021 PMC6474359

[5] Taylor KR , Costanzo AE, Jameson JM. Dysfunctional γδ T cells contribute to impaired keratinocyte homeostasis in mouse models of obesity. J Invest Dermatol. 2011;131:2409–2418. 21833015 10.1038/jid.2011.241 PMC3213272

[6] Gray EE et al IL-17-committed Vγ4+ γδ T cell deficiency in a spontaneous Sox13 mutant CD45.1 congenic mouse substrain protects from dermatitis. Nat Immunol. 2013;14:584–592. 23624556 10.1038/ni.2585 PMC3660499

[7] Harper EG et al Th17 cytokines stimulate CCL20 expression in keratinocytes in vitro and in vivo: implications for psoriasis pathogenesis. J Invest Dermatol. 2009;129:2175–2183. 19295614 10.1038/jid.2009.65 PMC2892172

[8] Zhou S , Li Q, Wu H, Lu Q. The pathogenic role of innate lymphoid cells in autoimmune-related and inflammatory skin diseases. Cell Mol Immunol 2020;17:335–346. 32203190 10.1038/s41423-020-0399-6 PMC7109064

[9] Carolan E et al Altered distribution and increased IL-17 production by mucosal-associated invariant T cells in adult and childhood obesity. J Immunol. 2015;194:5775–5780. 25980010 10.4049/jimmunol.1402945

[10] Cai Y et al Pivotal role of dermal IL-17-producing γδ T cells in skin inflammation. Immunity. 2011;35:596–610. 21982596 10.1016/j.immuni.2011.08.001 PMC3205267

[11] Taylor KR , Mills RE, Costanzo AE, Jameson JM. γδ T cells are reduced and rendered unresponsive by hyperglycemia and chronic TNFα in mouse models of obesity and metabolic Disease. PLoS One. 2010;5:e11422. 20625397 10.1371/journal.pone.0011422 PMC2896399

[12] Nielsen MM et al IL-1β-dependent activation of dendritic epidermal T cells in contact hypersensitivity. J Immunol Baltim MD 1950. 2014;192:2975–2983. 10.4049/jimmunol.1301689 PMC4020443 24600030

[13] MacLeod AS et al Dendritic epidermal T cells regulate skin antimicrobial barrier function. J Clin Invest 2013;123:4364–4374. 24051381 10.1172/JCI70064 PMC3784546

[14] Havran W , Allison J. Origin of Thy-1+ dendritic epidermal cells of adult mice from fetal thymic precursors. Nature. 1990;344:68–70. 1968230 10.1038/344068a0

[15] Barbee SD et al Skint-1 is a highly specific, unique selecting component for epidermal T cells. Proc Natl Acad Sci USA 2011;108:3330–3335. 21300860 10.1073/pnas.1010890108 PMC3044407

[16] Jameson J et al A role for skin gammadelta T cells in wound repair. Science. 2002;296:747–749. 11976459 10.1126/science.1069639

[17] Sharp LL , Jameson J, Cauvi G, Havran WL. Dendritic epidermal T cells regulate skin homeostasis through local production of insulin-like growth factor 1. Nat Immunol. 2005;6:73–79. 15592472 10.1038/ni1152

[18] Daniel T et al Regulation of the postburn wound inflammatory response by gammadelta T-cells. Shock Augusta Ga. 2007;28:278–283. 17545947 10.1097/shk.0b013e318034264c

[19] Liu Y et al Single-cell profiling reveals divergent, globally patterned immune responses in murine skin inflammation. iScience. 2020;23:101582. 33205009 10.1016/j.isci.2020.101582 PMC7648132

[20] Mabuchi T et al CCR6 is required for epidermal trafficking of γδ T cells in an IL-23-induced model of psoriasiform dermatitis. J Invest Dermatol. 2013;133:164–171. 22895364 10.1038/jid.2012.260 PMC3511632

[21] Schutyser E , Struyf S, Van Damme J. The CC chemokine CCL20 and its receptor CCR6. Cytokine Growth Factor Rev. 2003;14:409–426. 12948524 10.1016/s1359-6101(03)00049-2

[22] Anderson LS et al CCR6+ γδ T cells home to skin wounds and restore normal wound healing in CCR6-deficient mice. J Invest Dermatol. 2019;139:2061–2064.e2. 30935975 10.1016/j.jid.2019.02.032 PMC6708754

[23] Wu C , Chen X, Shu J, Lee C-T. Whole-genome expression analyses of type 2 diabetes in human skin reveal altered immune function and burden of infection. Oncotarget. 2017;8:34601–34609. 28427244 10.18632/oncotarget.16118 PMC5470994

[24] Gray EE , Suzuki K, Cyster JG. Identification of a motile IL-17 producing γδ T cell population in the dermis. J Immunol Baltim MD 1950. 2011;186:6091. 10.4049/jimmunol.1100427 PMC3098921 21536803

[25] Gargas S , Bshara-Corson S, Cruz M, Jameson J. Isolation and analysis of mouse and human skin γδ T cells. Curr Protoc Immunol. 2019;127:e92. 31763791 10.1002/cpim.92

[26] Tan L et al Single-cell transcriptomics identifies the adaptation of Scart1+ Vγ6+ T cells to skin residency as activated effector cells. Cell Rep. 2019;27:3657–3671.e4. 31216482 10.1016/j.celrep.2019.05.064

[27] Chi X et al RORα is critical for mTORC1 activity in T cell-mediated colitis. Cell Rep. 2021;36:109682. 34525365 10.1016/j.celrep.2021.109682

[28] Komori HK et al Cutting edge: dendritic epidermal γδ T cell ligands are rapidly and locally expressed by keratinocytes following cutaneous wounding. J Immunol Baltim MD 1950. 2012;188:2972–2976. 10.4049/jimmunol.1100887 PMC3311739 22393149

[29] Girardi M et al Regulation of cutaneous malignancy by gammadelta T cells. Science. 2001;294:605–609. 29685949

[30] Nielsen MM , Witherden DA, Havran WL. γδ T cells in homeostasis and host defence of epithelial barrier tissues. Nat Rev Immunol. 2017;17:733–745. 28920588 10.1038/nri.2017.101 PMC5771804

[31] Nie Z et al c-Myc is a universal amplifier of expressed genes in lymphocytes and embryonic stem cells. Cell. 2012;151:68–79. 23021216 10.1016/j.cell.2012.08.033 PMC3471363

[32] Zhu B et al Early growth response gene 2 (Egr-2) controls the self-tolerance of T cells and prevents the development of lupus like autoimmune disease. J Exp Med. 2008;205:2295–2307. 18779345 10.1084/jem.20080187 PMC2556781

[33] Zhang B , Jiao A, Dai M, Wiest DL, Zhuang Y. Id3 restricts γδNKT cell expansion by controlling Egr2 and c-Myc activity. J Immunol Baltim MD 1950. 2018;201:1452–1459. 10.4049/jimmunol.1800106 PMC6103809 30012846

[34] Wang R et al Genetic and pharmacological inhibition of the nuclear receptor RORα regulates TH17 driven inflammatory disorders. Nat Commun. 2021;12:76. 33397953 10.1038/s41467-020-20385-9 PMC7782731

[35] Liu Z et al Dendritic epidermal T cells facilitate wound healing in diabetic mice. Am J Transl Res. 2016;8:2375–2384. 27347345 PMC4891450

[36] Rees PA , Greaves NS, Baguneid M, Bayat A. Chemokines in wound healing and as potential therapeutic targets for reducing cutaneous scarring. Adv Wound Care. 2015;4:687–703. 10.1089/wound.2014.0568 PMC4620529 26543682

[37] Gómez-Melero S , Caballero-Villarraso J. CCR6 as a potential target for therapeutic antibodies for the treatment of inflammatory diseases. Antibodies. 2023;12:30. 37092451 10.3390/antib12020030 PMC10123731

[38] Liu Z et al Defects in dermal Vγ4 γ δ T cells result in delayed wound healing in diabetic mice. Am J Transl Res. 2016;8:2667–2680. 27398150 PMC4931161

[39] Chodaczek G , Toporkiewicz M, Zal MA, Zal T. Epidermal T cell dendrites serve as conduits for bidirectional trafficking of granular cargo. Front Immunol. 2018;9:1430. 29988392 10.3389/fimmu.2018.01430 PMC6023976

