# Supplementary material for: Impact of obesity on the CCR6-CCL20 axis in epidermal γδ T cells and IL-17A production in murine wound healing and psoriasis
Source: J Immunol. 2025 Jan 23;214(1):153–66. doi: 10.1093/jimmun/vkae011 (PMC11844138; doi:10.1093/jimmun/vkae011)
Supplement: vkae011_Supplementary_Data [file vkae011_supplementary_data.zip › JIMMUN-24-00153-s03.pdf]

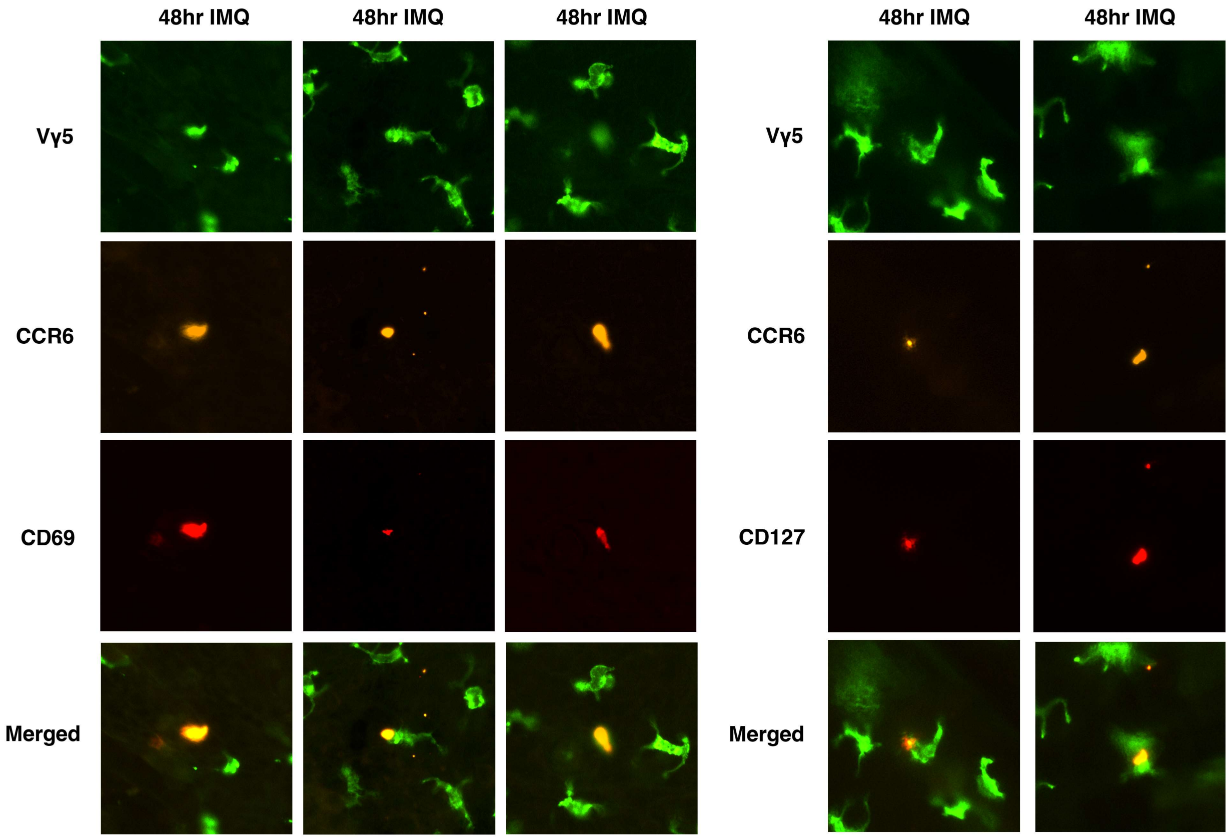

Supplemental Figure 1: Detection of Vγ5 T cells expressing CCR6, CD69, and CD127 in the epidermis of IMQ-treated mice.

Representative immunofluorescent images of epidermal sheets from B6 mice 48 hours after (IMQ) treatment. (Left panels) In IMQ-treated mice, 38 images were analyzed (n=5 mice), 93.75% of CCR6+ cells co-expressed CD69. In control mice, 25 images were analyzed (n=4 mice), with 0% co-expressing CD69.

(Right panel) In IMQ-treated mice, 28 images were analyzed (n=4 mice), 90.9% of CCR6+ cells co-expressed CD127. In control mice, 25 images were analyzed (n=4 mice), with no cells expressing CCR6 or CD127.
