# Supplementary material for: Impact of obesity on the CCR6-CCL20 axis in epidermal γδ T cells and IL-17A production in murine wound healing and psoriasis
Source: J Immunol. 2025 Jan 23;214(1):153–66. doi: 10.1093/jimmun/vkae011 (PMC11844138; doi:10.1093/jimmun/vkae011)
Supplement: vkae011_Supplementary_Data [file vkae011_supplementary_data.zip › JIMMUN-24-00153-s02.docx]

October 1, 2024

Dr. Gail Bishop

Editor-in-chief
The Journal of Immunology Editorial Office

Dear Dr. Bishop:

Please accept our resubmission of the manuscript, titled “Impact of Obesity on the CCR6-CCL20 Axis in Epidermal γδ T Cells and IL-17A Production in Murine Wound Healing and Psoriasis” *William Lawler, Tanya Castellanos, Emma Engel, Cristian R. Alvizo, Antolette Kasler, Savannah Bshara-Corson, and Julie M. Jameson*. We have added additional data and revised the manuscript according to the reviewer’s comments. Our changes are highlighted in yellow in the manuscript file and we have included a response to the reviewers. The findings described in this article will be of great interest to the Journal of Immunology readership and thus we wish to resubmit the article for publication.

Obesity is a pervasive problem in the United States with over one third of adults exhibiting a BMI over 30. It is clear that there are major immunological consequences that come from excess adipose tissue including barrier dysfunction in the skin, lung and intestine. Our previous studies have shown that intraepithelial lymphocytes in the skin lose the ability to participate in wound healing (Taylor et al, PLoS One, 2010, Taylor et al J. Invest. Derm., 2011, Park et al., 2017 J. Immunol). Here we have examined whether obesity impacts a subset of epidermal γδ T cells that express CCR6 and have the ability to produce IL-17 very early in psoriasis and wound repair. This study is appropriate for The Journal of Immunology as this is a hot topic in the field with recent reviews and articles in Cell Metabolism and Immunity among other journals. These findings have novel implications for the regulation of a specific population of IL-17-producing epidermal γδ T cells during skin damage and inflammation. In addition, the myc signaling pathway has not previously been correlated with epidermal γδ T cells in psoriasis as a mechanism for increasing IL-17.

All authors concur with this submission. The material in this study has not been previously published and is not under consideration elsewhere. We are willing to pay page charges in the case that the manuscript is approved for publication. Please do not hesitate to contact me if additional information is required.

Thank you for your consideration.

Sincerely,

Julie Jameson, PhD

Director of the Graduate Program in Biology

Co-Director of the Summer Scholars Program

Professor, Dept. of Biological Sciences

California State University San Marcos

Science Hall 1, Room 317

333 S. Twin Oaks Valley Rd.

San Marcos, CA 92096

(760) 750-8274

[jjameson@csusm.edu](mailto:jjameson@csusm.edu)
